# piRT-IFC: Physics-informed real-time impedance flow cytometry for the characterization of cellular intrinsic electrical properties

**DOI:** 10.1038/s41378-023-00545-9

**Published:** 2023-06-08

**Authors:** Xiaofeng Luan, Pengbin Liu, Di Huang, Haiping Zhao, Yuang Li, Sheng Sun, Wenchang Zhang, Lingqian Zhang, Mingxiao Li, Tian Zhi, Yang Zhao, Chengjun Huang

**Affiliations:** 1 Institute of Microelectronics of the Chinese Academy of Sciences, Beijing, China; 2 University of Chinese Academy of Sciences, Beijing, China; 3 State Key Laboratory of Computer Architecture, Institute of Computing Technology, Chinese Academy of Sciences, Beijing, China; 4 Cerebrovascular Diseases Research Institute, Xuanwu Hospital of Capital Medical University, Beijing, China

**Keywords:** Biosensors, Microfluidics

## Abstract

Real-time transformation was important for the practical implementation of impedance flow cytometry. The major obstacle was the time-consuming step of translating raw data to cellular intrinsic electrical properties (e.g., specific membrane capacitance C_sm_ and cytoplasm conductivity σ_cyto_). Although optimization strategies such as neural network-aided strategies were recently reported to provide an impressive boost to the translation process, simultaneously achieving high speed, accuracy, and generalization capability is still challenging. To this end, we proposed a fast parallel physical fitting solver that could characterize single cells’ C_sm_ and σ_cyto_ within 0.62 ms/cell without any data preacquisition or pretraining requirements. We achieved the 27000-fold acceleration without loss of accuracy compared with the traditional solver. Based on the solver, we implemented physics-informed real-time impedance flow cytometry (piRT-IFC), which was able to characterize up to 100,902 cells’ C_sm_ and σ_cyto_ within 50 min in a real-time manner. Compared to the fully connected neural network (FCNN) predictor, the proposed real-time solver showed comparable processing speed but higher accuracy. Furthermore, we used a neutrophil degranulation cell model to represent tasks to test unfamiliar samples without data for pretraining. After being treated with cytochalasin B and N-Formyl-Met-Leu-Phe, HL-60 cells underwent dynamic degranulation processes, and we characterized cell’s C_sm_ and σ_cyto_ using piRT-IFC. Compared to the results from our solver, accuracy loss was observed in the results predicted by the FCNN, revealing the advantages of high speed, accuracy, and generalizability of the proposed piRT-IFC.

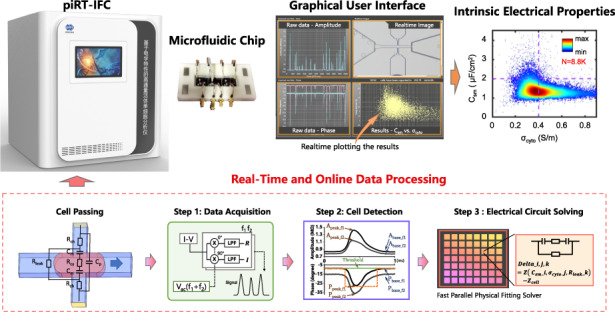

## Introduction

Electrical properties are important biophysical properties of single cells that are emerging as biomarkers for cell status characterization^1,2^ and cell classification^3,4^ in a label-free and noninvasive manner. At present, single-cell electrical property analysis has gained momentum in the fields of cell physiological activity^5–7^, disease diagnosis^8,9^, cancer aggressiveness^10,11^, drug screening^12–14^, and so on. As cellular intrinsic electrical properties, the specific membrane capacitance (C_sm_) and cytoplasm conductivity (σ_cyto_) are independent of cellular size and measuring system parameters^15^, enabling comparative analysis of results obtained with different methods or contributed by different research groups. Moreover, with the benefit of decoupling other cellular properties or measuring system parameters, C_sm_ and σ_cyto_ performed more effectively for cell classification than primitive impedance features^9,16^ and enabled the exploration of additional implied relations between different cellular inherent biophysical properties^17^.

Impedance flow cytometry (IFC) techniques are powerful tools for high-throughput electrical property characterization at the single-cell level^18,19^ and are widely used for blood cell differentiation^20^, phenotypic assays^21^, and morphology quantification^22^. With the emergence of applications of cellular electrical detection in fundamental life science and drug assessment research, the demand for real-time data analytic strategies for IFC is becoming increasingly prominent^23,24^. Some application scenarios, such as real-time monitoring^6,25,26^ and active sorting^27,28^ (e.g., electrical properties being used to trigger downstream cell isolation), have highlighted an urgent need for real-time data processing in the whole workflow from the original signal acquisition to biophysical parameter extraction. The development of real-time impedance flow cytometry is a matter of great urgency^29^. Several efforts have been made to develop real-time impedance flow cytometry^12,30^. These real-time IFCs were mainly based on phenomenal electrical signal variations (differential impedance amplitude and phase changes, termed opacity or electrical diameter) rather than cellular intrinsic electrical properties, thus resulting in relatively low accuracy and dependency on the instruments^31–34^. Due to the time-consuming process of solving the equivalent electrical circuit models for a large number of cells (i.e., >100k cells), RT-IFC based on cellular intrinsic electrical properties is still challenging^9^.

With significant progress in neural network (NN) frameworks, NN-aided techniques have been increasingly applied in IFC applications, in which an advanced NN algorithm was used to accelerate the data process for real-time monitoring purposes^29,35^. With the assistance of NNs, impressive performances for rapidly predicting cellular intrinsic electrical properties were recently reported^16,36^.

Despite the powerful ability of NN-aided approaches for rapidly mapping raw data to cellular features, the main drawback of such approaches was the dependency of their predicting performance on the quality and volume of training datasets. As a black box, the neural network encoded the mapping relationships between input and output into its connections with training processes, which made the predicting capability dominated by the accuracy, completeness, and adequacy of information embedded in the training datasets^37^. However, for IFC techniques, big datasets for training a network to completely respect the underlying physical principles to predict cellular intrinsic electrical properties cannot be easily obtained^38^. A compromise solution for NN-aided IFC to improve the accuracy is to characterize a few cells using the traditional physical model fitting solver, train the networks with the characterized cells, and then perform follow-up tests on the rest of the cell samples. In this case, the accuracy and generalizability ability of the neural network-based approach should be addressed when testing an unfamiliar cell sample without data prepared for pretraining. That solution might be reasonable for some particular applications, such as blood classification and counting^36,39^. However, in broader scenarios, such as clinical diagnosis or new drug discovery studies, cell samples cannot always be preacquired, which may result in a low-quality training dataset for the NN-aided IFC. In those scenarios, exploring physics-informed approaches to decouple the dependence on the pretraining process is essential^37^.

**Fig. 1:**
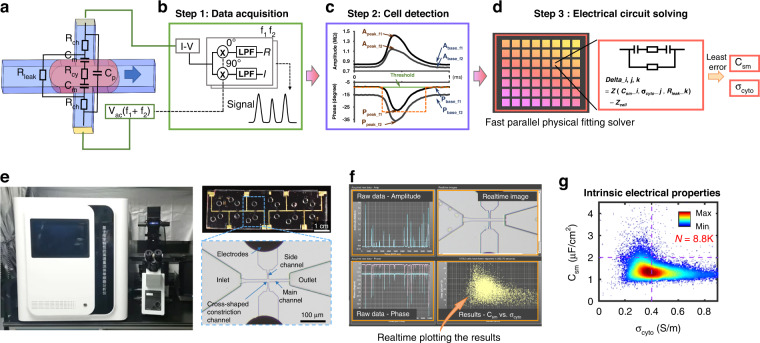
Schematic, workflow, images, and output results of piRT-IFC. **a** Schematic of the crossing constriction channel-based impedance flow cytometry; **b** Raw impedance data acquisition with hardware and software facilities; **c** Cell detector used to detect cells and extract their impedance feature vectors; **d** Fast parallel physical fitting (FPPF) solver for obtaining intrinsic electrical properties C_sm_ vs. σ_cyto_; **e** Photograph of the piRT-IFC and microscopic image of the microfluidic chip embedded with a cross-shaped constriction channel; **f** Interface for users to control and watch output results; **g** Scatter plot of intrinsic electrical properties of single cells’ C_sm_ vs. σ_cyto_ obtained in a five-minute cycle

Here, we proposed a fast parallel physical fitting (FPPF) solver to obtain single-cell intrinsic electrical properties from raw impedance data. Based on the solver and the hardware of the impedance flow cytometry reported in our previous study^40^, we implemented physics-informed real-time impedance flow cytometry (piRT-IFC) for online data acquisition, cell detection, and intrinsic electrical property solving (Fig. 1). Furthermore, with piRT-IFC, the speed, accuracy, and generalizability ability of the FPPF solver and the NN-aided predictor were evaluated and compared to reveal the advantages of using the physics-informed solution.

## Results and discussion

### Principle and setup

Figure 1 shows the principle of the developed piRT-IFC. Briefly, the microfluidic chip with a cross-shaped constriction channel was designed to form dynamic transient mega-ohm resistance cell sealing when single cells were continuously aspirated to pass through the main constriction channel. Figure 1A shows the equivalent electrical circuit model when the cell was sealed in the cross-shaped constriction channel. The signal source, lock-in amplifier, and data acquisition program were simultaneously used to acquire impedance changes between the side constriction channel when cells pass (Fig. 1B). From the acquired raw impedance data frames, the real-time cell detector detected single cells immediately and extracted eight impedance feature parameters (Fig. 1C). Finally, the fast parallel physical fitting (FPPF) solver rapidly translated the eight parameters to single cells’ C_sm_ and σ_cyto_ (Fig. 1D).

We integrated the hardware, the microfluidic chip (Fig. 1E), and the developed real-time cell detector, the FPPF solver, the user interface (Fig. 1F), into a platform called physics-informed real-time impedance flow cytometry (piRT-IFC). While working, the recorded impedance signals and the real-time images were displayed simultaneously while the single cells passed through the constriction microchannel. Meanwhile, the characterized single cells’ C_sm_ and σ_cyto_ were plotted online in real-time. Finally, single cells’ C_sm_ and σ_cyto_ could be exported for deeper analysis after a characterization cycle (Fig. 1G).

### Real-time characterization performance

The performance of the developed piRT-IFC was verified by characterizing the electrical properties of single cells from the A549 and 293T cell lines. Video S1 recorded the screen of the piRT-IFC in run time, demonstrating the ability to characterize the single cells’ intrinsic electrical properties in real time with high throughput. Video S2 recorded the entire 5-min cycle for characterizing 12,440 cells in real time by the piRT-IFC and was replayed at tenfold speed, indicating that the piRT-IFC was stable for a long working time. Those screen recording videos confirmed that the piRT-IFC’s processing speed satisfied the requirement for stable real-time characterization of single cells’ intrinsic electrical properties in a throughput of more than 100,000 cells/h^40^.

**Fig. 2:**
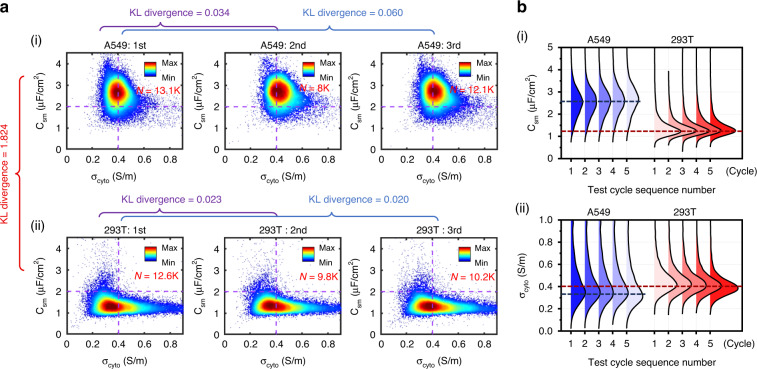
Performance evaluation results of the piRT-IFC. **a** Scatter plots of intrinsic electrical properties (C_sm_ vs. σ_cyto_) of single cells from A549 and 293T cell lines characterized in real-time and **b** the ridgeline plots of the probability density distribution of C_sm_ (i) and σ_cyto_ (ii) of A549 (blue) and 293T (red) cells, respectively. The dotted lines represented the position of the peaks of distributions for A549 (blue) and 293T (red) cells. N refers to the number of cells

The scatter plots in Fig. 2A show the single-cell intrinsic electrical properties distribution (C_sm_ vs. σ_cyto_) of A549 and 293T cells characterized in the first three cycles. Electrical property distributions measured at different times had a high similarity for the same sample, for either A549 or 293T cells. We employed KL divergence to quantify the distribution differences and found that the KL divergence < 0.1 between two cycles of A549 vs. A549 or 293T vs. 293T, which means that there was little difference between the results characterized at different cycles for cells from the same cell line. In comparison, the KL divergence was 1.824 between two cycles of A549 vs. 293T, which was much larger than that for the same cell line ( < 0.1). The significant difference between the electrical properties of A549 and 293T cells also proved that the developed piRT-IFC could distinguish different samples. The ridgeline plots in Fig. 2B displayed the probability density distributions of C_sm_ and σ_cyto_ of 100,902 single A549 and 293T cells characterized in real-time for 10 cycles (5 cycles for each of A549 or 293T) within 50 min. Figure 2B(i) indicated that the peaks of the probability density distribution of A549 cells’ C_sm_ in 5 cycles were all located around 2.6 μF/cm^2^ (the blue dotted line), while the peaks of distributions of 293T cells’ C_sm_ in 5 cycles were all located around 1.2 μF/cm^2^ (the red dotted line). Figure 2B(ii) indicated that the peaks of distributions of A549 cells’ σ_cyto_ in 5 cycles were all located around 0.3 S/m (the blue line), while the peaks of distributions of 293T cells’ σ_cyto_ in 5 cycles were all located around 0.4 S/m (the red line). The above results indicated the temporal stability of the piRT-IFC over multi-cycles.

### Performance of the FPPF solver compared with other methods

We further assessed the performance of the proposed fast parallel physical fitting (FPPF) solver by comparing it with a previously reported traditional solver and fully connected neural network (FCNN) predictor in terms of data processing speed and accuracy. We selected the raw data of A549 cells recorded in cycles in Section 2.2 and then solved (or predicted) single cells’ C_sm_ and σ_cyto_ with the traditional solver we previously reported^40^, the proposed FPPF solver, and the FCNN, respectively. For the FCNN, we trained the network with the raw data (input) and single cells’ C_sm_ and σ_cyto_ (output) characterized by the traditional solver from the first cycle of A549 cells and then predicted the single cells’ C_sm_ and σ_cyto_ from raw data from other cycles of A549 cells.

**Fig. 3:**
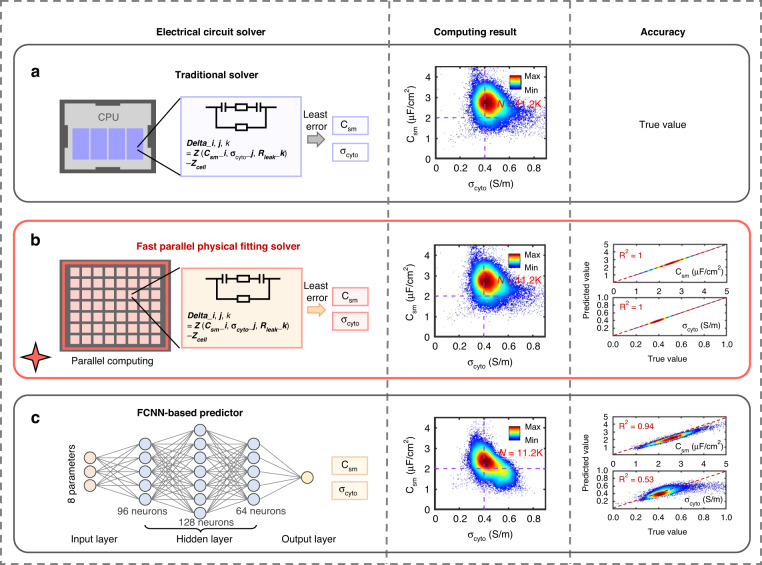
Comparison of different data translation strategies. The characterization results obtained by the traditional solver were regarded as true values **a**. Then, the same raw data were processed by two real-time strategies: the proposed FPPF solver **b** and the reproduced FCNN predictor **c**. The single-cell electrical properties obtained from these three solvers were exhibited using scatter plots. The accuracy was expressed as the agreement of the results obtained through real-time solver **b**, **c** versus the true values obtained through **a**. N refers to the number of cells

Figure 3 shows the schematic diagrams and processed results of the last cycle by the three methods. The characterization results obtained by the traditional solver were regarded as true values (Fig. 3A). The characterization results obtained by the other two real-time strategies were compared to the true values to evaluate the accuracy. To evaluate the agreement between each of the two real-time strategies and the traditional solver, we calculated the R-square values of C_sm_ and σ_cyto_ between the real-time strategies and the true values from the traditional solver. Figure 3B shows that both C_sm_ and σ_cyto_ solved by the FPPF and traditional solver were highly linear (y = x) with R^2^ = 1 because we used the same equivalent electrical circuit model in the two solving processes. Figure 3C shows that the prediction accuracy of the FCNN predictor was lower than that of the FPPF solver, with R^2^ = 0.94 for C_sm_ and R^2^ = 0.53 for σ_cyto_. Moreover, a more focused trend was found for the results predicted by the FCNN, indicating that some biases might appear in the results located at the peripheral regions with sparse points. Such biases might be attributed to inherent data imbalance in the real world and might cause low accuracy for predicting the C_sm_ and σ_cyto_ of rare abnormal cells (e.g., circulating tumor cells) with NN-based strategies in further applications.

**Fig. 4:**
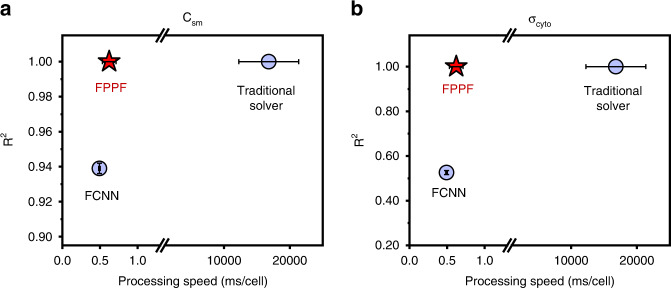
Comparisons of the three methods in terms of processing speed and accuracy. **a** Scatter plot for R^2^ of C_sm_ vs. processing speed. **b** Scatter plot for R^2^ of σ_cyto_ vs. processing speed. The results were statistically obtained with all cycles and represented as the mean ± SD

Considering that translating raw impedance data to single cells’ intrinsic electrical properties was the most time-consuming step in the entire data processing procedure, the translation speeds of the three methods were quantified and compared. We recorded the entire elapsed times using the three methods for translating all single cells ( ~ 10,000 cells) in one cycle of A549 cells and statistically analyzed them. Figure 4 shows that the processing speeds were 16,800 ± 455, 0.62 ± 0.09, and 0.49 ± 0.01 ms/cell for the traditional solver, the proposed FPPF solver, and the FCNN predictor, respectively. From those results, we found that the FPPF solver achieved 27000-fold acceleration compared to the traditional solver and had a speed comparable to that of the FCNN predictor of less than 1 ms/cell. Notably, the processing time of 0.62 ms for one cell was less than the frame interval time of 2 ms, which meant that we could translate in real time.

Moreover, we calculated the two methods’ R-square values of cellular C_sm_ and σ_cyto_ in every cycle and obtained the averages of the R^2^ of cellular C_sm_ (Fig. 4A) and σ_cyto_ (Fig. 4B) by statistically analyzing the results for the last four cycles of A549 cells. Figure 4 shows the comparisons among the three methods in terms of the accuracy and data processing speed, indicating that the FPPF solver had higher accuracy than the FCNN predictor and was faster than the traditional solver, indicating that the proposed FPPF solver was a better candidate for real-time single-cell intrinsic property characterization.

### Real-time characterization of cellular electrical properties in dynamic changes

To demonstrate the generalizability of the physics-informed method, we selected the neutrophil degranulation cell model to represent unfamiliar cells. In this section, we first characterized the intrinsic electrical properties (C_sm_ and σ_cyto_) of cells from the control and drug-treated groups. After treatment with cytochalasin B (CB) and N-Formyl-Met-Leu-Phe (fMLP), HL-60 cells (a neutrophil cell line) underwent degranulation, in which the cellular dynamic behaviors changed in less than one second and continued for ten minutes^41^. With piRT-IFC, we continuously characterized the intrinsic electrical properties of single HL-60 cells from the control and drug-treated groups in real time for 30 and 25 min.

**Fig. 5:**
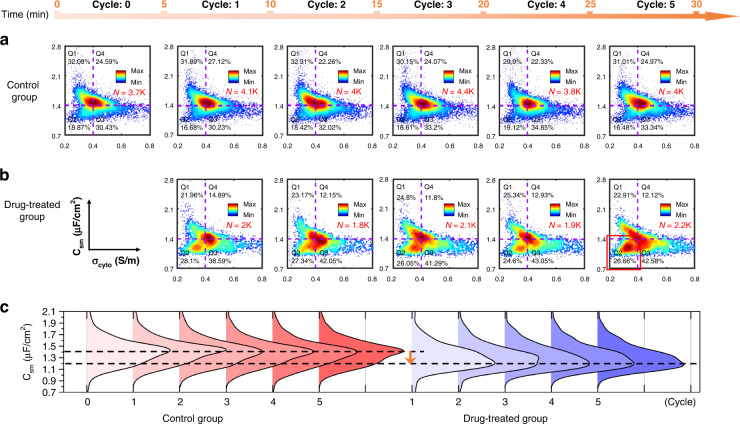
The intrinsic electrical properties of HL-60 cells from the control and drug-treated groups. Scatter plots of intrinsic electrical properties (C_sm_ vs. σ_cyto_) of single cells from the **a** control and **b** drug-treated groups in 5-min cycles in 30 min. A colormap described the data point density, and two purple dotted lines (placed at C_sm_ = 1.4 μF/cm^2^ and σ_cyto_ = 0.4 S/m) divided the panel into four quadrants of Q1–Q4, with the proportion labeled. **c** Ridgeline plots of C_sm_ density distribution in cycles of the cells from control (red) and drug-treated (blue) groups. N refers to the number of cells

Figure 5A, B show scatter plots of the C_sm_ vs. σ_cyto_ of single cells from the control and drug-treated groups in 5-min cycles, respectively. As a reference, two perpendicular purple dotted lines (the horizontal line represents C_sm_ = 1.4 μF/cm^2^, and the vertical line represents σ_cyto_ = 0.4 S/m) were placed to divide the diagrams into four quadrants Q1–Q4, with the cell proportion annotated in each quadrant. The C_sm_ vs. σ_cyto_ of cells from the control group were found to be invariantly focused on the intersection of the purple dotted lines in the six 5-min cycles (Fig. 5A). In contrast, the electrical properties of cells from the drug-treated group emerged as continuous dynamic changes over five 5-min cycles (Fig. 5B). Especially in Q2, a subcluster appeared with dynamic variation over time. In addition, compared to the control group, the proportions of the drug-treated group in Q2 and Q3 increased, revealing that the C_sm_ of drug-treated group cells tended to decrease over time when suffering the degranulation process.

Figure 5C shows the density distributions of single cells’ C_sm_ in cycles with ridgeline plots, indicating the variation tendency in single cells’ C_sm_ from the control (red) to drug-treated (blue) group. With reference to the above black dotted line placed at C_sm_ = 1.4 μF/cm^2^, distributions of C_sm_ of cells from the control group were found to be strongly consistent. In contrast, the drug-treated group’s peaks roughly migrated to the vicinity of the below black dotted line placed at C_sm_ = 1.2 μF/cm^2^. Notably, the shapes of the ridgelines of the five cycles are different, indicating the dynamic migration of subgroups in cycles of the drug-treated group. With two black lines, the variation tendency in C_sm_ from the control group to the drug-treated group could be observed, labeled by an orange arrow. The variation tendency and dynamics might present challenges for translating methods with low generalizability.

### Performance of the FPPF solver compared with the FCNN predictor

We performed a comparative study on the accuracy variations when characterizing familiar and unfamiliar cell samples to reveal the inherent generalizability of the FPPF solver and FCNN predictor. Herein, the raw data recorded in Section 2.4 was reused to obtain single cells’ intrinsic electrical properties (C_sm_ vs. σ_cyto_) with the FCNN predictor offline. To simulate the real usage scenario of the neural network, we used the data of the first three cycles from the control group as the training dataset to train the FCNN. We then used the trained FCNN to predict the intrinsic electrical properties of cells of the remaining three cycles from the control group and the first three cycles from the drug-treated group. Considering the apparent migration in cellular C_sm_ from the control group to the drug-treated group, we mainly examined the performance of the FCNN for predicting C_sm_. The C_sm_ distributions of cells from the control and drug groups in the last cycle obtained with the FPPF solver and FCNN predictor were then compared for analysis.

**Fig. 6:**
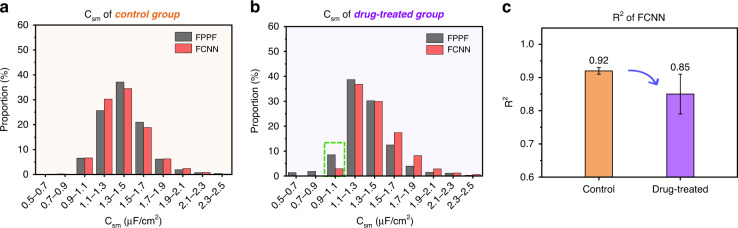
Performance comparison between the FPPF solver and FCNN predictor for characterizing the C_sm_ of cells from the control and drug-treated groups. **a** The C_sm_ distributions of the last cycle in the control group characterized by two methods. **b** The C_sm_ distributions of the last cycle in the drug-treated group characterized by two methods. The green dotted box indicates a significant difference between the proportion of the FPPF solver and the FCNN predictor. **c** Histogram of R^2^ of cells’ C_sm_ from the control group and drug-treated group predicted with FCNN, represented as the mean ± SD

Figure 6A shows the C_sm_ distribution of cells in one cycle from the control group, indicating the relative consistency of the distribution obtained by the two methods. In contrast, Fig. 6B shows the C_sm_ distribution of cells in one cycle from the drug-treated group, with an apparent difference between the two methods in the distribution zone of 0.9–1.1 μF/cm^2^ (labeled using a green dotted box). Compared with Fig. 6A, B, the distribution bias in the drug-treated group might result from the distribution information of the drug-treated group when using the FCNN predictor. Interestingly, speculating based on the variation trends from the control to the drug-treated group, the subgroup cells whose C_sm_ were located around C_sm_ = 1 μF/cm^2^ might be active cells undergoing degranulation.

Figure 6C shows the averages of R^2^ for predicting the C_sm_ of HL-60 cells in the three cycles from the control and drug-treated groups. For the control group, cellular C_sm_ showed better linearity (y = x) with R^2^ = 0.92 ± 0.01, indicating that the results predicted by the FCNN agreed well with the results from the FPPF solver. However, the R^2^ of the drug-treated group was 0.85 ± 0.06, of which the significantly decreased average indicated lower accuracy, and the significantly increased standard deviation showed lower stability than the control group.

Therefore, for invariant cell samples, which could be partly characterized to obtain their electrical properties as the training dataset before prediction, the FCNN predictor had comparable accuracy and stability to the FPPF solver. However, when facing unfamiliar cells, the proposed FPPF solver performed better in terms of accuracy and stability than the FCNN predictor, exhibiting its inherent good generalizability achieved by using explicit physics laws.

With the accumulation of data in the future, we also expect a physics-informed neural network, embedding physics laws into the network framework, to emerge as another candidate for the next-generation RT-IFC^37^.

## Materials and methods

### Setup of real-time impedance flow cytometry

In this study, we mainly focused on the advanced data processing methodology and tried to address the challenge of translating the raw impedance data to cellular intrinsic electrical properties in real time. Based on the hardware of the impedance flow cytometry we previously reported^17,40^, the setup comprised the following five essential components: a cross-shaped constriction channel-embedded microfluidic chip, a dual-frequency AC sinusoidal signal source (WF1974, NF Corporation, JAPAN), a lock-in amplifier (model 7270, signal recovery, USA) for impedance monitoring, a pneumatic controller (PACE5000, GE Druck, Leicester, UK), and the software we studied in this work. The detailed manufacturing protocol and materials for the microfluidic chip are presented in the supplementary methods.

The front part of the software for graphical user interface, hardware control, and raw data acquisition was programmed with the LabVIEW platform (National Instruments, USA). The backbone of the software for data processing was hybrid programmed with Python (python.org), CUDA C + + (NVIDIA, USA), Intel Math Kernel Library (Intel, USA), and PyTorch (CUDA version, pytorch.org). The two parts were data-linked with local socket communication. Moreover, the software was run locally on a computer with the operating system Windows 10 (Microsoft, USA) and a hardware configuration including a microprocessor (Core i9-12900, Intel, USA), 64 GB of memory, and a graphic card (RTX3090, NVIDIA, USA).

### Implementation of real-time cell detector

As the first step of real-time data processing for impedance flow cytometry, in this study, we need to optimize the time for detecting every single-cell event to less than 1 ms. Thanks to the dynamically high-resistance sealing with the constriction channel, the distinct impedance changes were measured when cells passed through the constriction channel of the microfluidic chip, resulting in the need for only a simple threshold comparison to check out the occurrence of the single-cell event. The threshold was obtained by performing two substeps of kernel density estimation. First, we used a Gaussian kernel density estimation to obtain the potential impedance baseline in the latest 2 ms data frame. Then, we introduced the near-neighbor estimation among the baselines of the latest and historical frames to obtain the accuracy baseline to avoid threshold drifting in the case of potential temporary cell clogging during several frames. Once the single-cell events were detected by comparing the impedance data of every data frame with the threshold, eight impedance parameters were extracted for every cell, including the amplitude and phase for the peaks and baselines of the impedance stream in two frequencies of 100 and 180 kHz, for further processing steps. The above computing process was optimized as vector computing in multithreading with the support of the Intel Math Kernel Library.

### Implementation of fast parallel physical fitting solver

In this study, we obtained the intrinsic properties from the impedance profiles online using a fast parallel physical fitting (FPPF) solver. Because of the noisy impedance data acquired in the practical measuring process, we could not obtain the cellular intrinsic electrical properties C_sm_ and σ_cyto_ by directly analyzing the equivalent circuit model. We used the least error-fitting method in our previously reported work^40^. With the four baseline parameters we extracted by the cell detector, the resistance (R_ch_) and parasitic capacitance (C_p_) of the microchannel were obtained. According to the equivalent circuit model (Fig. 1A), the cell impedance (Z_cell_) can be obtained by decoupling R_ch_ and C_p_ from the total impedance (Z_total_). With the four peak parameters, Z_cell_ was further translated to C_sm_ and σ_cyto_. In the fitting process, we selected 256, 128, and 128 data points of C_m_, R_cyto_, and R_leak_ by linearly dividing the ranges from the minimum and maximum values of each parameter, respectively. The three parameters are determined by least error fitting:

$$\begin{array}{ll}{\rm{Delta}}{{\_}}{\rm{i}},{\rm{j}},{\rm{k}}=\,{\sum}_{{f=f}_{1}}^{{f}_{2}}\left\{\right.\left|{\mathrm{Re}}\left[{Z}_{f}({C}_{{sm}}{{\_}}i,{\sigma }_{{cyto}}{{\_}}j,{R}_{{leak}}{{\_}}k)\right]-{\mathrm{Re}}[{Z}_{f}{{\_}}{cell}]\,\right|\\ \qquad\qquad\qquad+\left|{Im}[{Z}_{f}({C}_{sm}{{\_}}i,{\sigma }_{{cyto}}{{\_}}j,{R}_{{leak}}{{\_}}k)]-{Im}[{Z}_{f}{{\_}}{cell}]\right|\left\}\right.\end{array}$$

To speed up the fitting process, we proposed a “run once and cached” strategy to compute the impedances in two frequencies of potential 8,388,608 circuits. With this strategy, while computing the first cell, the impedances of enumerated circuits with potential parameters were computed in parallel in less than one second with the GPU and cached in the GPU memory as a look-up table. After that, while computing the remaining cells, computing processes were turned to memory operations, whose operation times were independent of the physical model complexity and might be optimized to less than one millisecond per cell. Then, we used GPU-accelerated parallel additions and subtractions in the GPU memory. Finally, we adopted a GPU-accelerated MapReduce to rapidly find the best-fitting parameters with the least error for every cell.

### Comparative study with the neural network predictor

For a comparative study, we implemented a fully connected neural network (FCNN)^16^ in our IFC system to predict intrinsic electrical properties from the eight impedance parameters for each cell event obtained by the real-time cell detector. The FCNN included five layers: the input layer for inputting eight impedance parameters for every cell, the output layer for outputting cellular C_sm_ or σ_cyto_, and the three hidden layers for learning the mapping relationship between the input and output data. Note that C_sm_ or σ_cyto_ was separately trained and predicted in two networks with the same structure since we found this approach to work better than using the structure to output the two parameters together. The mean square error (MSE) function for computing the difference between the ground-truth values and predicted values was used as the loss function, and the stochastic gradient descent (SGD) optimizer was utilized to supervise training the FCNN. The FCNN was trained with a learning rate of 1e-3, batch size of 32, and epoch number of 50. The ground truth dataset of the network was obtained using the traditional model fitting method for intrinsic properties and manual labeling of the cell types. When tracking the processing speed, the FCNN was also trained to predict the R_leak_ to match the functional ability of the solvers. Thus, the operation of the three FCNNs in sequence to predict the three parameters for one cell was used to calculate the processing speed.

### Statistics and analysis

The Kullback‒Leibler (KL) divergence metric was employed to quantify the similarity and difference between the two distributions. KL divergence was represented as D_KL_(P||Q), which was calculated by the following formula:

$${D}_{{KL}}(P{\rm{||}}Q)=\,\mathop{\sum }\limits_{i}^{n}P({x}_{i}){\rm{log }}\left(\frac{P({x}_{i})}{Q({x}_{i})}\right)$$

Generally, the smaller D_KL_(P||Q) is, the greater the similarities between the distributions of Q and P.

Besides, the additional descriptions of “Materials”, “The Fabrication of the Microfluidic Chip”, and “Cell culture, Preparation and Characterization Operation” are collected in the electronic supplementary material.

## Conclusions

In this work, we proposed a new fast parallel physical fitting (FPPF) solver, which was proven to have the speed of 0.62 ms/cell to translate the raw impedance data vector to cellular intrinsic electrical properties, achieving ~ 27000-fold acceleration and the same accuracy compared to the traditional solver. Based on that, we implemented piRT-IFC and validated its performance by the real-time detection, translation, and output of the cellular intrinsic electrical properties of approximately 100,000 single cells in 50 min. We also compared the performance between the FPPF solver and the commonly used FCNN predictor for characterizing intrinsic electrical properties (C_sm_ and σ_cyto_) of A549 cells, HL-60 cells, and drug-treated HL-60 cells, which were selected to represent the unfamiliar cells that lacked preacquired data. Compared with the FCNN predictor, the proposed FPPF solver showed advantages in accuracy, generalizability, and needlessness of pretraining.

## Supplementary information

Supplemental Material

Video S1

Video S2
