# Supplementary material for: piRT-IFC: Physics-informed real-time impedance flow cytometry for the characterization of cellular intrinsic electrical properties
Source: Microsyst Nanoeng. 2023 Jun 8;9:77. doi: 10.1038/s41378-023-00545-9 (PMC10250341; doi:10.1038/s41378-023-00545-9)
Supplement: Supplementary file 1 — Supplemental Material [file 41378_2023_545_MOESM1_ESM.docx]

**piRT-IFC: A physics-informed real-time impedance flow cytometry for the characterization of cellular intrinsic electrical properties**

Xiaofeng Luan ^a,b^, Pengbin Liu ^a,b^, Di Huang ^c^, Haiping Zhao ^d^, Yuang Li ^a,b^, Sheng Sun ^a,b^, Wenchang Zhang ^a^, Lingqian Zhang ^a^, Mingxiao Li ^a^, Tian Zhi ^c,*^, Yang Zhao ^a,*^, Chengjun Huang ^a,b,*^

a Institute of Microelectronics of the Chinese Academy of Sciences, Beijing 100029, China

b University of Chinese Academy of Sciences, Beijing 100049, China

c State Key Laboratory of Computer Architecture, Institute of Computing Technology, Chinese Academy of Sciences, Beijing, 101125, China

d Cerebrovascular Diseases Research Institute, Xuanwu Hospital of Capital Medical University, Beijing 100053, China

### Materials

Unless otherwise stated, all reagents for cell culture were purchased from Thermo Fisher Scientific Inc. (Waltham, MA, USA). Specifically, relevant reagents used in cell culture include RPMI-1640 Media (GIBCO, Life Technologies Corp., USA), DMEM Media (GIBCO, Life Technologies Corp., USA), Fetal Bovine Serum (FBS) (GIBCO, Life Technologies Corp., USA), Phosphate Buffer Saline (PBS) (GIBCO, Life Technologies Corp., USA), Penicillin–Streptomycin (GIBCO, Life Technologies Corp., USA), 0.25% Trypsin (GIBCO, Life Technologies Corp., USA). Cytochalasin B (CB) (MACKUN, Macklin Inc., China), N-Formyl-Met-Leu-Phe (fMLP) (Sigma, Sigma-Aldrich, USA) was used for HL-60 degranulation model. Materials used for microfluid chip fabrication include SU-8 photoresist (MicroChem Corp., USA) and 184 silicone elastomer (Dow Corning Corp., USA).

### The fabrication of the microfluidic chip

The microfluidic chip was bonded with a PDMS cover and a glass substrate. The PDMS cover with the crossing constriction channel embedded was replicated from a dual-layer mold, in which the constriction channel layer with a height of 10 µm and the cell loading channel layer with a height of 30 µm were fabricated using deep silicon etching and SU-8 photoresist molding, respectively. Meanwhile, the glass substrate with planer gold electrodes on top was micro-fabricated with a conventional lift-off procedure. After being treated with oxygen plasma, the PDMS cover was aligned and bonded to the glass slide.

### Cell Culture, Preparation and Characterization Operation

All cell lines were purchased from the Infrastructure of Cell Line Resources and cultured in a cell incubator (3111, Thermo Scientific, USA) at 37 °C in 5% CO_2_. Specifically, the lung cell line A549 and the human acute promyelocytic leukemia cells HL-60 were cultured with RPMI-1640 media, while the human embryonic kidney cell line 293T was cultured with DMEM media. Both media were supplemented with 10% FBS and 1% Penicillin–Streptomycin. The A549 or 293T cells were trypsinized using 0.25% trypsin (GIBCO, Life Technologies Corp., USA) and resuspended to the single cell suspension with a concentration of 3.3×10^6^ cells/mL for the experiment. Before every 5-minute cycle of single-cell electrical characterization, 15 μL of cell suspension with about 50,000 A549 or 293T cells contained was loaded to the inlet of the microfluidic chip.

For the dynamic physiological changes of single cells, the drug model of HL-60 degranulation was established by being treated with CB and FMLP. HL-60 cells in the logarithmic growth phase were collected and followed by centrifuging to remove the supernatant. The collected cells were re-suspended in RPMI-1640 media for a concentration of 2×10^6^ cells/ml and then were randomly divided into two aliquots. One aliquot was characterized without treatment and regarded as the control group, while the other aliquot was pretreated with 5 μg/mL CB for 2 min and then 1 mM FMLP for 3 min (drug-treated group), and was immediately followed by single-cell electrical characterization.

After loading the prepared cell sample to the inlet of the microfluidic chip pre-filled with medium, we applied negative pressure to the outlet to drive the single cells passed through the crossing-shaped constriction channel and simultaneously acquired the impedance changes. This basic characterization operation was the same as our previous work described [17]. Notably, the negative pressure was set as 10 kPa in section A549 and 293T cells characterization experiment, the same as we previously reported [17]. Meanwhile, considering the HL-60 cells might become easy to break after treatment of CB, we applied 3 kPa of the negative pressure during the section of HL-60 cells characterization experiments.
